# Age-related mTOR in gynaecological cancers

**DOI:** 10.18632/aging.101190

**Published:** 2017-02-28

**Authors:** Preety Bajwa, Subhransu S. Sahoo, Pradeep S. Tanwar

**Affiliations:** Gynaecology Oncology Group, School of Biomedical Sciences and Pharmacy, University of Newcastle, Callaghan, New South Wales, Australia

**Keywords:** aging, mTOR, cancer

Aging is an uninvited sequel of organismal growth and the ultimacy of which is death. At the molecular level, organismal aging is a conversion from cellular quiescence to senescence state. Age is a major risk factor for many diseases including diabetes, obesity, neurodegenerative disorders, and cancer. Other consequences of aging are not defined by diseases, but rather considered ‘stages of life’, because they normally occur in everyone, such as menopause in females. However, deregulated female reproductive cycles (early menarche and/or late menopause) lead to hormonal imbalance and cancer at later stages of life. Endometrial and ovarian cancers are the most frequently diagnosed cancers in aged women and account for significant mortality. The incidence of both these cancer increases with advancing age, peaking around 60 to 70 years of age [1, 2]. Up to 80% of such patients are found to have genetic aberrations in the members of the PI3K-mTOR (phosphoinositide 3-kinase-mammalian target of rapamycin) pathway [3, 4]. mTOR signaling is important in regulating the cell cycle of the majority of proliferating cells, where homeostasis is maintained by either cell division or quiescence [5]. In contrast, hyperactive mTOR signaling causes cellular hypertrophy, which alters homeostasis, leading to cellular senescence, cancer and age-related diseases [5].

Recently, we demonstrated that with advancing age, female mice and humans develop endometrial and ovarian surface epithelial (OSE) cell hyperplasia, papillary growth and inclusion cysts [4, 6]. Histopathological examination of these abnormal growths revealed expression of bonafide markers of human ovarian cancer precursor lesions, Pax8 and Stathmin 1, and concurrent elevated expression of pS6 protein (a downstream target of the mTOR pathway) compared to young mice and humans [6]. Genetic deletion or overexpression of *PTEN* (a negative regulator of mTOR signaling) significantly altered the age-associated changes in female mice and human reproductive tract organs [4]. Furthermore, pharmaco-logical suppression of the mTOR pathway using rapamycin treatment significantly reduced both endometrial and OSE hyperplasia in aged mice [4, 6]. Taken together, these observations established that the age-associated pathological changes in the female reproductive tract organs are driven by aberrant mTOR signaling.

**Figure 1:**
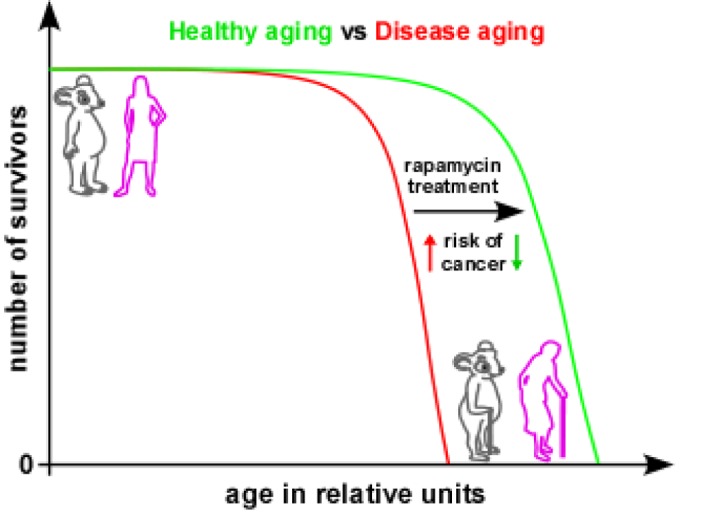
Hypothetical model of mTOR-driven cancer in aged women A potential therapeutic opportunity for the treatment of gynaecologic cancers is outlined. In post-menopausal aged women, rapamycin treatment can suppress hyperactive mTOR signalling in ovarian and uterine epithelium, potentially promoting healthy aging.

Cancer is an age-related disease and any lifestyle changes or interventions that slow aging also delay cancer [7]. Many independent studies have already demonstrated the effectiveness of an anti-aging drug, rapamycin, in many species. In model organisms, rapamycin prolongs lifespan and delays cancer, even when calorie restriction does not. The TOR signaling is a major evolutionarily conserved player in longevity regulation as pharmacological modulation of this pathway extends lifespan in flies, worms, yeast and mice. Under these circumstances, our study provides evidence that rapamycin treatment inhibits mTOR-driven precancerous lesions in the female reproductive tract organs and may also extend lifespan or promote healthy aging in post-menopausal women. Our study highlights that even rapamycin treatment in genetically heterogeneous, 9 months-old mice (equivalent to ∼50 years age of human) significantly suppresses mTOR activity and gynaecologic cancer lesions. Thus, if rapamycin can rescue aging phenotypes in cancer-prone transgenic mice, it is predictable that rapamycin intervention in post-menopausal women should delay age-related cancers and promote a healthy life. Nevertheless, many questions remain to be answered. In aged mice and women, why is overactive mTOR specific to a few cell types of the reproductive tract organs? Is there any link between unopposed estrogen signaling and the mTOR pathway that leads to hyperplastic epithelium of the uterus and ovary? Moreover, how can we use this knowledge to treat cancer? Our studies, therefore, open up an avenue to explore the mTOR pathway as a target for prevention and/or treatment of gynaecological cancers.
